# Many faces of FoMO: A qualitative in-depth investigation of context-specific experiences, emotions, and coping strategies

**DOI:** 10.1371/journal.pone.0330978

**Published:** 2025-09-02

**Authors:** Diana Jaworska

**Affiliations:** 1 Faculty of Psychology, University of Warsaw, Warsaw, Poland; 2 Faculty of Economic Sciences, University of Warsaw, Warsaw, Poland; Neighborhood Physical Therapy, UNITED STATES OF AMERICA

## Abstract

This study examines how individuals experience fear of missing out (FoMO) across online and offline contexts and the strategies they use to cope. Sixteen individual in-depth interviews (IDIs) with participants aged 18–35 revealed three categories of FoMO: (1) FoMO related to social media, (2) offline FoMO intensified by social media, and (3) solely offline FoMO. Each category features distinct emotional and behavioral responses, with universal coping strategies like distraction, self-reflection, and support-seeking, alongside social media-specific methods such as limiting information access and engaging in a social media detox. This study uniquely encompasses multiple FoMO contexts within a single framework, providing a more comprehensive view of FoMO’s emotional and behavioral impacts. Findings broaden the understanding of FoMO beyond social media, offering valuable insights for mental health professionals and social media platforms in addressing its diverse effects on well-being.

## Introduction

In today’s hyper-connected world, people are constantly exposed to endless opportunities and experiences they could pursue. With the rise of technology and social media, nearly everything feels within reach, creating a persistent pressure to stay informed and engaged. However, as access to these experiences grows, so too does the anxiety of missing out. This phenomenon, known as Fear of Missing Out (FoMO), has become increasingly prevalent. FoMO refers to the pervasive feeling that others are enjoying rewarding experiences, gaining knowledge, or acquiring possessions from which one is excluded [1]. In an age where substantial parts of our lives are broadcasted online, understanding FoMO has become more relevant than ever.

As interest in FoMO grows, researchers have increasingly turned their attention to this phenomenon, leading to a growing number of studies [2,3]. However, much of this research has focused narrowly on FoMO in the context of social media, often linking it to issues like social media addiction and behaviors such as “phubbing” [4,5]. While valuable, this focus risks oversimplifying FoMO by confining it to the digital realm. Importantly, the term FoMO was coined before the rise of social media, indicating that it encompasses a broader range of experiences, both online and offline [6,7]. Despite this, the differences between online and offline FoMO, and how these various contexts might be related to distinct emotions or behaviors, remain underexplored.

Furthermore, as FoMO becomes more widespread, its potential consequences, such as depressive symptoms, decreased life satisfaction, and lower mood, underscore the importance of identifying coping mechanisms [1; 8–10]. Though some studies have addressed this, they too tend to focus primarily on online-related FoMO [11,12]. It remains unclear whether individuals employ different coping strategies in response to FoMO in offline settings compared to digital ones.

Given these gaps, a qualitative approach is essential to fully capture the complexity of FoMO across various contexts. Most research on the topic consists of cross-sectional surveys, with very few qualitative studies available [2]. Scholars have called for more qualitative techniques in FoMO research [2]. While a few in-depth qualitative studies exist, they tend to focus on a single context, such as social media-related FoMO [11] or consumer behavior [13,14]. There is currently a lack of research that explores how FoMO unfolds across multiple life contexts and how individuals navigate and cope with these experiences holistically. Qualitative research is particularly valuable in this area because it enables a deeper understanding of how people make sense of their FoMO experiences in different settings. It allows for rich, nuanced insights that quantitative methods alone may overlook, especially when exploring emotions, meanings, and coping processes.

The study is guided by research questions focusing on the contexts in which FoMO arises, the emotions associated with these experiences, and the coping strategies people adopt in response.

## Literature review

### Theoretical backgrounds of FoMO

Although research on FoMO is expanding, its theoretical foundations remain relatively limited (for a review, see [2]). One of the most widely referenced frameworks is Self-Determination Theory (SDT) [15], which posits that individuals are motivated by three core psychological needs: autonomy, competence, and relatedness. FoMO can arise when individuals feel deprived of experiences or opportunities that satisfy these needs, often prompting increased social media use as a way to compensate [1]. SDT has been extensively applied in studies on social media-related FoMO, with many researchers interpreting FoMO in this context as a response to unmet psychological needs [2,3]. However, SDT has also been applied beyond the digital realm, with some studies exploring FoMO in other contexts, such as alcohol-related coping [16]. Despite these efforts, SDT has predominantly been used to explain FoMO in relation to social media. The theory’s assumptions are well-supported by evidence linking FoMO to increased social networking site use [4]. However, one limitation of SDT in this context is that it primarily frames FoMO as a motivational deficit, focusing on internal need frustration, and may underemphasize external social cues or comparative processes that trigger FoMO.

Social Comparison Theory (SCT) [17] offers another useful lens for understanding FoMO. SCT proposes that people assess their self-worth by comparing themselves to others. When individuals see others engaging in rewarding activities or achieving success one is missing out on, FoMO may be triggered, resulting in negative emotions such as anxiety, envy, and a sense of inadequacy [18–20]. This theory has primarily been used to explain FoMO in the context of social media [20,21], where constant exposure to curated online lives can intensify upward comparisons. In contrast to SDT, which emphasizes unmet internal needs, SCT views FoMO as a reaction to perceived external discrepancies between the self and others.

Both SDT and SCT predominantly focus on FoMO in the context of social media, though they can also be extended to offline experiences. In contrast, Zhang et al. [22] offer a more universal framework for understanding FoMO. They argue that FoMO is triggered by psychological threats to the self-concept, which refers to “the totality of an individual’s thoughts and feelings about themselves as an object” [23]. According to this perspective, FoMO has both personal and social dimensions: personal FoMO involves missing out on opportunities that enhance one’s private self, while social FoMO pertains to experiences that enhance one’s public self. This model broadens the understanding of FoMO, suggesting that it can arise in any context, whether online or offline. Unlike SDT and SCT, which tend to emphasize either psychological needs or comparative processes, Zhang et al.’s model integrates self-concept as a core mechanism, acknowledging both personal and interpersonal drivers of FoMO. This theoretical pluralism invites further discussion about whether FoMO is best understood as a unitary construct or a context-dependent phenomenon with multiple emotional and cognitive pathways.

### Contexts of experiencing FoMO

Fear of Missing Out is often closely associated with social media use, as most studies on this phenomenon focus specifically on that context (see reviews by [2,3]). Research supports the idea that FoMO may drive excessive or problematic social media and smartphone use [4,9,24,25]. However, the term FoMO predates the rise of social media, suggesting that it may encompass a broader range of experiences [6,7]. Indeed, some studies indicate that FoMO is not exclusive to social media contexts [26]. People can experience FoMO regardless of how they become aware of missed opportunities, although social media may amplify this awareness [26]. This raises the question of whether FoMO is fundamentally a technologically mediated phenomenon, or whether social media merely acts as a catalyst for a more general psychological experience.

Despite this, only a few definitions capture FoMO in more general terms [22,27]. For instance, Zhang et al. [22] define FoMO as “a fear of potential negative consequences from inaction on perceived opportunities”. With such an approach, FoMO can apply to both online and offline settings. However, it remains unclear whether FoMO experiences differ depending on the context or if they are fundamentally the same across various settings. This ambiguity points to a theoretical gap: while some models treat FoMO as a stable trait, others suggest it is context-sensitive and situational. These perspectives are rarely compared or integrated, limiting our understanding of how context may shape the emotional and behavioral consequences of FoMO.

Outside the realm of social media, FoMO has been explored in various contexts, including the workplace [28], consumer decision-making [29], media consumption [30], sports viewing [31], cinema attendance [32], news and information updates [33], event attendance [34], and social interactions [26]. In each of these studies, FoMO within a specific context was linked to some negative outcomes. However, most of these investigations are limited in scope, focusing on one context at a time without offering broader comparisons of FoMO experiences across multiple settings. As a result, the literature remains fragmented, with little insight into whether FoMO is experienced similarly across domains or whether certain contexts (e.g., work vs. leisure) elicit more intense or distinct emotional responses.

A recent review by Groenestein et al. [3] highlights ongoing confusion in the literature regarding the conceptualization of FoMO. While researchers generally agree that FoMO involves unpleasant feelings about missing out on experiences, there is still debate about the range of emotions that accompany it. This ambiguity is significant given the multifaceted nature of FoMO, which can manifest in both social and personal dimensions, as Zhang et al. [22] suggest. These different dimensions may evoke different emotional responses and experiences, yet this issue remains underexplored. This lack of clarity calls for a more nuanced investigation into how FoMO is felt and processed across different life domains, and whether emotional consequences vary depending on these contexts. Based on this gap, the following research questions were formulated:

**RQ1.** In what contexts do people typically experience FoMO?

**RQ1a.** What emotions are commonly associated with the experience of FoMO in different contexts?

To better understand this complexity, a qualitative, in-depth approach is needed to explore and compare FoMO across various contexts. This is particularly important because, regardless of the domain in which FoMO is studied, it is consistently linked to negative outcomes, such as negative affect and emotional distress [26]. Consequently, it is crucial to examine how people cope with FoMO and whether their coping strategies vary depending on the context in which FoMO is experienced.

### Coping with FoMO

The negative consequences of FoMO have led researchers to explore various strategies for combating this feeling. However, much like FoMO research itself, most of these studies focus specifically on social media-related FoMO. Many interventions treat FoMO as a form of social media addiction or compulsive use, with strategies aimed at reducing problematic social media behavior [12]. These approaches often involve limiting social media use or implementing a “social media detox” [11,12,35]. Some interventions even propose redesigning social media platforms to reduce exposure to information, block certain apps for set periods, or provide self-closing chats and alert messages [12]. A similar approach is the FoMO Reduction Method (FoMO-R) [11], which outlines six stages in a self-help guide. While these strategies have proven effective, they remain focused solely on FoMO within the digital context. Such approaches also tend to conceptualize FoMO as a maladaptive behavior requiring behavioral restriction, potentially overlooking deeper emotional or cognitive mechanisms behind the phenomenon.

Beyond FoMO-specific interventions, a broader review of problematic social media use suggests that therapy-based interventions, and full abstinence from social media, are more effective for improving well-being than simply limiting use [35]. These interventions, aimed at individuals with problematic social media habits, may also reduce FoMO by encouraging participants to reevaluate life priorities, engage in other activities, and minimize social comparison and envy [36,37]. In contrast, limiting social media use alone has been found to be less effective. This raises questions about whether coping with FoMO requires external behavior change or internal psychological shifts, such as reappraising one’s needs or expectations, which is based on self-awareness.

Other successful approaches include group counseling and cognitive restructuring, shown to reduce FoMO in social media contexts, though these techniques can be applied more broadly [38]. Similarly, mindfulness practices have been suggested as a remedy for FoMO, helping individuals experience the “joy of missing out” (JOMO) and promoting greater well-being [39,40]. Although these studies focus on social media, mindfulness techniques could potentially be effective for managing FoMO in offline contexts as well. However, the extent to which these strategies generalize across different FoMO contexts such as career, education, or relationships, remains largely unexplored. Moreover, some interventions promote acceptance and reframing (e.g., JOMO), while others emphasize avoidance or digital withdrawal, pointing to divergent assumptions about whether FoMO should be eliminated, regulated, or transformed.

Further insights come from research showing that emotion regulation mediates the relationship between FoMO and problematic internet use [41]. Enhancing emotional regulation could therefore be another avenue for reducing FoMO, even though this study focuses specifically on internet use. These findings highlight the importance of understanding FoMO not only as a behavioral response but also as an emotional and cognitive experience shaped by individual regulation strategies.

Despite the focus of these studies on social media FoMO, it remains unclear whether FoMO in other contexts triggers different coping mechanisms. Moreover, while these interventions test the effectiveness of various strategies, little is known about how people naturally cope with FoMO or whether they need to employ specific strategies at all. Understanding the intuitive strategies people use and their perceptions of these approaches is crucial for designing interventions that truly address FoMO across different contexts. This lack of evidence also invites reconsideration of whether all instances of FoMO require coping, or whether some may be motivating, or even adaptive depending on the context. This leads to the following research question:

**RQ2.** What strategies do people use to cope with FoMO?

## Current study

Despite the growing body of research on FoMO, several important issues remain underexplored. The majority of FoMO research has focused on social media [4,5], which, while prevalent, is not the only context in which FoMO is experienced. Exploring FoMO in offline settings is crucial, as it may evoke different emotions and behaviors. Fewer studies have examined FoMO across multiple contexts [29,31], and this gap needs to be addressed to better understand how FoMO manifests beyond the digital realm.

Moreover, given the negative consequences associated with FoMO, understanding how individuals cope with it is of paramount importance. While some studies have investigated coping strategies [11,12], these are almost exclusively limited to social media contexts. The question of how people cope with other forms of FoMO, and whether coping strategies differ across contexts, remains largely unexplored.

This study aims to fill these gaps by investigating individuals’ experiences of FoMO in both online and offline contexts, and the strategies they use to cope with it.

To explore these issues, we employed individual in-depth interviews (IDIs). This method was chosen for two key reasons. First, IDIs are particularly effective for uncovering complex psychological mechanisms and emotions, some of which may be unconscious [42]. Given our goal of understanding FoMO in various contexts, this approach allowed for richer, more detailed insights from each participant compared to other research methods. Second, due to the personal and potentially sensitive nature of the topic, IDIs provided a private and comfortable environment, reducing potential barriers to open discussion that could arise in group settings. Moreover, recent reviews on FoMO research highlight that most studies rely on cross-sectional survey designs [3,4], which may not provide the depth of understanding necessary to fully grasp individuals’ lived experiences.

### Method

#### Design.

The individual in-depth interviews (IDIs) were conducted to explore the contexts in which participants experience FoMO, the emotions associated with it, and the strategies used to manage it. A phenomenological approach was adopted to gain insight into participants’ lived experiences of FoMO. The study took place from June 24 to July 31, 2024, with interviews conducted online using the Google Meet platform. Only audio recordings were made, and all transcripts were anonymized during transcription to remove any identifying information. Before participating, respondents were informed about the study’s purpose, process, and voluntary nature, and they were assured they could withdraw at any time. Each participant received a 50 PLN voucher for an online multibrand store as compensation for their participation.

#### Participants.

A total of 16 individual in-depth interviews (IDIs) were conducted. The study employed a purposeful sampling strategy, aimed at capturing a diverse range of perspectives relevant to the research questions. Participants were intentionally selected to reflect variation in gender, age, and trait FoMO levels – all factors considered relevant to the experience of FoMO. Recruitment took place via advertisements in social media groups, where a link to a screening questionnaire was shared (see S1 File). In total, 83 individuals completed the questionnaire. From this pool, invitations were sent to 20 participants who met the selection criteria, of which 16 agreed to participate. The selection criteria included a balanced gender ratio (approximately 50% male), an age range of 18–35, and a balanced distribution of FoMO traits (50% high FoMO and 50% low FoMO; see Table 1). Participants were located in various regions of Poland.

**Table 1:** List of participants.

Participant	Trait FoMO level	Gender	Age
1	low (2.00)	F	19
2	low (2.00)	F	23
3	low (2.90)	F	28
4	low (2.40)	F	34
5	low (2.20)	M	20
6	low (2.20)	M	24
7	low (2.40)	M	28
8	low (1.51)	M	32
9	high (3.80)	F	21
10	high (4.30)	F	23
11	high (4.60)	F	30
12	high (4.00)	F	32
13	high (4.30)	F	20
14	high (3.20)	M	23
15	high (3.40)	M	32
16	high (4.70)	M	34

The final sample consisted of 9 women and 7 men, aged 18–35, as FoMO has been found to be most prevalent within this age group according to a Polish report on FoMO [43]. Participants’ FoMO traits were assessed using the FoMO Scale developed by Przybylski et al. [1], and a cutoff score of 3.00 was used to distinguish between high and low FoMO. This cutoff was chosen because 3.00 represents the median value of the scale, which ranges from 1 to 5. It reflects a conceptual midpoint and was consistent with both the median and mean observed in our screening sample (N = 83). Including trait-FoMO as a criterion enabled a comparison of FoMO experiences and coping strategies between individuals more and less prone to the phenomenon.

#### Data collection.

Each interview lasted approximately 90 minutes and followed a semi-structured format. This flexible approach allowed for adjustments to the questions and topics based on the flow of the conversation, ensuring that the interviews catered not only to the research objectives but also to the individual needs of each respondent. Before beginning the formal interview, the moderator briefly explained the purpose and relevance of the study to help establish trust and ensure participants felt comfortable sharing their experiences. The introductory portion of the interview focused on social media usage, serving as a warm-up. Data from this section was analyzed only if FoMO was spontaneously mentioned by the participant.

The main topics in the interview guide included: (a) associations with FoMO (including a projection technique exercise); (b) personal experiences of FoMO; (c) emotions accompanying FoMO (using Plutchik’s Wheel of Emotions); (d) methods of preventing FoMO; (e) strategies for coping with FoMO; and (f) general reflections on the phenomenon. The questions were developed based on a review of existing FoMO literature and guided by the study’s research questions, with the aim of capturing participants’ lived experiences and coping strategies. Theory-informed tools, such as Plutchik’s Wheel of Emotions and a projective technique, were incorporated to facilitate emotional reflection and support deeper insight into subjective experiences. The discussion guide and materials used in the study can be found in S2 and S3 Files. All interviews were conducted by a female interviewer with a background in psychology and experience in moderating qualitative interviews.

Data collection continued until thematic saturation was reached. Saturation was assessed iteratively throughout the data analysis process by monitoring whether new interviews continued to yield novel insights, codes, or themes. As data became increasingly repetitive and conceptually rich, it was determined that sufficient depth had been achieved to fully explore the research questions. This decision was guided by the principle that saturation depends more on the richness and depth of the data than on sample size alone [44].

#### Data analysis.

All interviews were transcribed in Polish by the moderator, and the transcripts were then double-checked for accuracy. The entire analysis process was conducted using the Polish-language material, which was the native language of both the author and the respondents. MAXQDA 2024 qualitative analysis software was used to process the data, applying thematic analysis to identify key themes [45]. The data analysis was conducted by the author. The initial codes were grouped into broader themes, and the organization of these codes and themes was refined in collaboration with a researcher holding a PhD in psychology. Peer debriefing was employed to review code definitions and the thematic structure, enhancing the credibility of the analysis. While the primary coding was performed by the author, peer input was used to evaluate the coherence and consistency of the emerging themes. A total of 653 statement excerpts were coded, creating 88 codes from five main areas: perception of FoMO; experiencing FoMO; emotions accompanying FoMO; strategies of dealing with FoMO; prevention methods. The code tree is presented in S4 File. The whole dataset (transcripts) can be found in OSF: https://osf.io/fnvdh/?view_only=134c46d48dd74dd484b623336270beb5.

#### Trustworthiness.

To enhance the trustworthiness of the study, several strategies were employed, following the established criteria [46]. Credibility was supported through prolonged engagement with participants, as interviews lasted approximately 1.5 hours and allowed for in-depth exploration rather than brief or surface-level questioning. Peer debriefing was used throughout the analysis process to critically examine emerging themes and reduce potential researcher bias. Reflexivity was maintained through ongoing self-reflection to acknowledge and manage the researcher’s positionality and assumptions. To ensure dependability, the coding process was clearly documented, including the development of codes, coding decisions, and analytic steps taken. Transferability was supported by providing thick descriptions of participant experiences and contextual details, allowing readers to assess the relevance of findings to other settings.

#### Ethical statement.

The study was approved by the Ethics Committee of the Faculty of Psychology at the University of Warsaw, Poland (decision no 09/04/2024/15). Participants received both written and verbal information about the study. This included a clear explanation that participation was voluntary, that they could withdraw at any time without consequences, and that their data would be kept confidential in compliance with ethical and data protection standards. Initial consent was expressed by participants in the screening questionnaire administered prior to the study, where they indicated their agreement with the study conditions. Before the main study commenced, verbal informed consent was obtained from all participants. This verbal consent was documented by the moderator, who recorded confirmation of consent in the study notes, without additional written documentation. The process for obtaining consent verbally, as described above, was explicitly reviewed and approved by the Ethics Committee.

To ensure privacy, quotes are presented only with gender, age, and FoMO level.

## Results

### Contexts of experiencing FoMO and emotions that accompany it

To investigate the contexts in which FoMO is experienced, participants were first asked to describe situations where they had personally felt FoMO. Since social media usage was introduced at the beginning of the interviews, it naturally emerged as a primary context in which FoMO can occur. Following the initial open-ended responses, participants were then presented with a structured question listing specific potential situations. They were asked to indicate whether or not these situations applied to them. The list of contexts included decision-making and consumer choices, work or study environments, and social interactions. When discussing each context, participants were also asked to reflect on the emotions and experiences associated with FoMO in that particular setting.

The discussed contexts were then analyzed and coded into common themes. Based on these contexts, they can be grouped into three main settings: (1) experiencing FoMO related to social media content; (2) experiencing FoMO related to offline situations, amplified by social media content; and (3) experiencing FoMO solely in an offline context.

#### FoMO related to social media content.

The main theme that emerged when discussing the contexts of experiencing FoMO was *social media*. However, it is important to note that FoMO related to social media was mentioned in two ways: (1) as the feeling of missing out on social media posts, and (2) as the feeling of missing out on experiences that occur offline but are shared on social media. The latter situation will be described in the next section. Here, we will focus on FoMO related to social media, specifically as the feeling of missing out on what is posted online.

***Constant pressure to stay connected.*** The fear of missing out on social media updates creates pressure to constantly check one’s phone. Participants noted that when they are unable to check social media in the moment, they feel a need to catch up later. In this context, some participants displayed compulsive tendencies toward checking social media. Additionally, trends such as current memes or updates from the lives of celebrities, influencers or friends were frequently mentioned. Participants expressed a need to keep up with these updates to avoid falling behind in comparison to their social groups. Falling behind could lead to exclusion from conversations, both online and offline, with peers.

*There were hundreds, thousands of profiles that I followed, telling myself that I had to keep up with the news from the world, because if I didn’t, I would miss out on something. And if I missed out on something, I wouldn’t be able to talk about it with my friends, and if I couldn’t talk about it with my friends, I would lose some great relationships.* [Man, 23, high FoMO]

***Need for information.*** Beyond social media trends, another frequently mentioned theme was the *need for information*. Participants described FoMO in this context as the fear of missing out on important, up-to-date information, such as news, often shared on social media or other online sources. Because this information changes so rapidly, participants felt pressured to constantly check what has been posted online.

***Emotional responses.*** Regarding the emotions associated with FoMO in this context, participants reported feelings of anxiety and irritation, especially when unable to check social media updates in real time. These emotions appeared tied to the perceived urgency of staying connected and informed. Shame and embarrassment (often framed as self-blame) were also common, arising when someone failed to stay up to date with online information. These emotions were more social in nature, reflecting fears of exclusion or judgment. Loneliness was mentioned when participants perceived themselves as falling behind or disconnected from peers.

On the other hand, curiosity was noted as a motivating emotion, which prompts the habit of checking social media content. Some also reported relief after checking updates, though this was more common among low FoMO participants, suggesting a lower emotional cost to engagement.

Although participants with high and low trait-FoMO described similar situations, those with high trait-FoMO tended to experience these events with greater emotional and behavioral intensity. This suggests that while the qualitative nature of FoMO experiences may be shared, trait-FoMO amplifies both the emotional and behavioral responses, potentially increasing the psychological impact of these moments.

*I mean, I wouldn’t call it any super anxiety, etc., but there’s just some discomfort that I feel when I’m not following what’s going on. Maybe it’s more subconscious than conscious sometimes, but it’s definitely present in my life.* [Woman, 19, low FoMO]

#### FoMO related to offline situations, amplified by social media content.

***Social interactions.*** The next set of situations where FoMO is experienced relates to offline events, but these feelings can be amplified by social media posts. The most common theme in this context was *social interactions*. When participants were asked directly whether they experience FoMO outside of social media, the most frequent response involved missing out on social activities, such as parties and gatherings. Participants noted that FoMO often originates offline but is intensified by seeing social media posts, such as photos from a party they were unable to attend. The underlying mechanism mentioned here is social comparison. As a result, participants reported feeling left out or as though they were falling behind socially. Their behavioral response to this type of FoMO was also to check social media.

While the original source of FoMO in these cases is offline, participants consistently described social media as intensifying emotional responses, particularly through processes of social comparison, perceived exclusion, and pressure to conform. This context elicited emotions that were rather interpersonal in nature, including loneliness, envy, and even guilt or self-blame.

*This feeling was rather supported by the fact that later I saw photos on social media from a given event and only then did I feel more strongly that I was missing out on something. But if there had been no mention in social media, I probably wouldn’t have felt it so much.* [Woman, 22, high FoMO]

Moreover, participants noted that what they see on *social media* often makes them feel as though they are missing out on opportunities and exciting experiences in real life. Social comparison was again a key factor. In addition to the pressure to stay updated, participants described feeling compelled to spend their time in ways that seemed more exciting or meaningful.

*I feel like I should do something, achieve something, not to spend time like that, sitting and using social media, but to go and do something. I have this feeling that there is quite a lot of pressure to spend time rather actively, and that you have to show that you are in so many places. So, this anxiety is more about being idle.* [Woman, 30, high FoMO]

***Culture and sports.*** Another theme that emerged in the context of FoMO was *culture and sports*, particularly in relation to one-time events. Participants frequently mentioned feeling like they were missing out on cultural events, such as concerts or sports games. This feeling was often intensified by seeing social media posts about the event. In terms of cultural experiences, participants also expressed frustration over missing out on new releases of movies, TV series, and books. They found it overwhelming to keep up with everything. Some participants reported feeling pressured to consume as much content as possible, driven by social comparison after seeing information about events on social media.

*I feel this way [FoMO] about cultural texts, for example. I really like reading and I have the feeling that there are a lot of very interesting books that I am missing out on, but which I somehow do not find on my way.* [Woman, 28, low FoMO]

***Online dating.*** A related theme that arose was *online dating*. Here, FoMO is amplified by a specific type of social media – dating apps. Some participants mentioned that the ease of seeing potential connections on dating apps made them feel like they were missing out on better opportunities. As a result, they felt the need to constantly check these apps to avoid missing the perfect match. Notably, this context was rarely mentioned spontaneously.

*Using Tinder when swiping left/right, I had the feeling that if I swiped for a while longer, the prince charming would appear. Or when the limit of likes was running out, I had to wait 8–12 hours and I thought that for sure in that time someone would create an account, then delete it, and that would be the one person for me that I’m goanna miss.* [Man, 23, high FoMO]

***Consumer choices.*** To a lesser extent, FoMO, though amplified by social media, was also reported in the context of *consumer choices*. This theme surfaced during the discussion after participants were directly asked about it, rather than being brought up spontaneously. Some participants acknowledged feeling left out when they saw new trends or products on social media, but this feeling was neither frequent nor intense for most.

***Emotional responses.*** In terms of emotions, participants highlighted socially driven feelings, such as fear of exclusion, loneliness, and envy, particularly in relation to missing social interactions or not keeping up with others’ lives. These emotions were often intensified by visual content on social media, which made absences more salient.

On the other hand, when comparing their lives to others, some participants also reported motivational emotions, such as inspiration to engage in similar experiences, suggesting that FoMO in this context can have a dual emotional valence. However, several participants described self-directed emotions, including self-blame, sadness, anxiety, restlessness, and anger, especially when they felt responsible for not participating or initiating social contact.

*There were situations when I blamed myself. Because I don’t like writing to people, I wrote to them little, and I blamed myself for that. Even though I may have seen them once or twice in my life, I blamed myself for not meeting them, for not writing to them. So, I thought about myself that I am a weak person because I miss out on great situations.* [Man, 23, high FoMO]

In terms of differences between individuals with high and low trait-FoMO, again the only distinction observed was the intensity of reported emotions, while the experiences and situations where FoMO was felt remained similar.

These findings point to FoMO as not only a reactive emotion but also a reflective one, triggered by perceived absence but deepened by self-evaluation.

#### FoMO related to an offline context.

The third setting of FoMO-related experiences involves situations where the feeling of missing out is tied to offline environments. Unlike FoMO triggered by social media or visible social activities, FoMO in offline contexts was often associated with long-term consequences and more future-oriented concerns, such as career advancement or life decisions. These experiences tended to involve achievement-related or existential pressures rather than immediate social exclusion.

***Work or study.*** The most frequently mentioned theme in this context was *work or study* settings. Participants described FoMO as the fear of missing important work-related updates or social events while being absent from work, school, or university. This feeling was more prominent among individuals with professional careers than among students. It is often related to the fear that during their absence, opportunities for career advancement, promotions, or networking may occur. As a result, participants reported feeling pressured to work harder and engage in more work-related activities to ensure they are not missing out or falling out of the loop. This pressure can also lead to behaviors such as checking work-related updates, like emails, even while on vacation, which can make it difficult to fully relax during time off. The behavioral responses to this feeling often include working more, taking on additional activities, and frequently checking work-related communications.

*When it comes to FoMO, when I was working, I had this unhealthy belief that this job was my entire personality and everything that was happening at work, I had to be up to date with, because I couldn’t be an employee who didn’t care about what’s happening at work. I thought that maybe I would take more shifts to find out what was going on* [Man, 23, high FoMO]

***Decision-making.*** The next theme related to FoMO in an offline context is *decision-making*. While participants were asked by the moderator about FoMO in terms of consumer behavior and decision-making, their responses suggest that FoMO related to consumer choices is more influenced by social media updates. In general, however, FoMO in decision-making appears to be more universal and connected to offline contexts. It is important to note that most participants did not mention this theme spontaneously but reflected on it only after being prompted. In the context of decision-making, some participants observed that they might feel FoMO in any situation involving choices, as making a decision inherently means missing out on the unchosen options. Interestingly, even when participants were satisfied with their choice, they still occasionally reported experiencing FoMO. This type of FoMO is linked to a sense of loss, which seems unavoidable. Additionally, some participants mentioned thinking about what they might have missed out on and considering alternative scenarios, which may involve counterfactual thinking. Notably, the decisions associated with this context tended to be more significant, such as career choices or where to live. Everyday decisions rarely evoked FoMO, and if they did, it was much less intense.

*I think the most striking example of this is my move from Krakow to another town. We decided to move to my hometown, which is much smaller. (…) We gained some things from it, but we lost others. And, of course, I have the feeling that, after my departure, there are a lot of interesting things happening in Krakow, which I miss out on. (…) I don’t regret this decision one bit, but emotionally there are thoughts that it was nice in Krakow (…).* [Woman, 28, low FoMO]

***Emotional responses.*** The emotional landscape of offline FoMO was marked by anxiety and stress, often tied to the fear of falling behind professionally or making suboptimal life choices. Participants described restlessness and a sense of pressure to engage in many activities (mostly work-related). In decision-related contexts, emotions such as regret, loss, and rumination were more common, highlighting a cognitive-emotional loop where FoMO is sustained by imagining better outcomes that were not chosen.

*I felt something like this when I wanted to be in two places at once, but I couldn’t for various reasons, and I had to choose. That’s when I feel like I’m losing something, that I was missing out on something.* [Woman, 23, high FoMO]

In terms of differences between individuals with high and low trait-FoMO, those with high trait-FoMO reported a wider range and greater intensity of emotions in the context of offline FoMO compared to those with low trait-FoMO. Instances of experiencing offline FoMO were also more frequent among individuals with high trait-FoMO, though participants in both groups admitted to experiencing it.

To summarize the emotional experiences associated with FoMO across the three discussed settings, individuals with high trait-FoMO tended to report more negative emotions, and these emotions were of greater intensity compared to those with low trait-FoMO. Conversely, individuals with low trait-FoMO were more likely to highlight the positive aspects of FoMO, such as the motivation it can provide for taking action. However, the range of situations in which people experienced FoMO was similar in both groups. This suggests that trait-FoMO may not only amplify the emotional intensity of FoMO but also shape how individuals evaluate and respond to it – socially, behaviorally, and emotionally.

**Fig 1:**
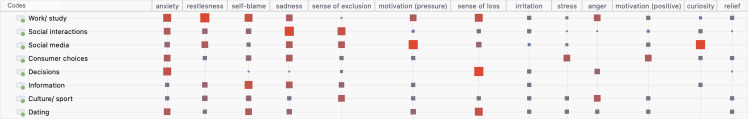
Proximity of codes (situations of feeling FoMO and emotions) in the same document*. * This figure depicts the number of times two codes were assigned at a maximum distance of 10 paragraphs. Notably, it does not show the precise relation between the two codes but only the proximity of its occurrence.

Fig 1 presents a summary of the situations in which FoMO is experienced and the emotions mentioned when discussing it, illustrating the proximity of codes from these two categories. It is important to note that the graph does not depict the exact relationship between these codes but rather shows the proximity of their occurrence.

### Strategies of dealing with FoMO

#### Methods of preventing FoMO.

Before discussing strategies people use to cope with FoMO, participants were asked whether they employed any methods to prevent FoMO from occurring in the first place. The majority admitted to not using any specific strategies aimed at preventing FoMO. This suggests that for many participants, FoMO is perceived as a reactive emotion rather than something that can be proactively managed. Notably, those who reported using preventive strategies were predominantly individuals with lower trait-FoMO, suggesting that these participants may engage in more anticipatory regulation or may be less emotionally reactive to potential social comparisons.

***Online methods.*** Most prevention strategies were focused on FoMO related to social media. These included limiting the content that appears on social media platforms by selectively choosing whom to follow. Another common approach was setting time limits for social media use.

*I think one way is a lack of contact with the sources that cause FoMO. (…) When it comes to social media, maybe it’s just avoiding spaces that might cause FoMO. And honestly, when I started talking about it loudly, maybe that’s why I don’t have Instagram. Because I’m simply cutting myself off from the possibility of seeing the content that would make me feel uncomfortable.* [Woman, 28, low FoMO]

***Offline methods.*** In offline contexts, participants more often described cognitive or emotional strategies, such as accepting FoMO as a natural part of life or managing expectations (as a way to reduce the emotional impact of potential missed experiences). These reflect more internal, meaning-focused forms of regulation. This process of acceptance seemed to depend on recognizing and naming the experience of FoMO, suggesting that a level of emotional literacy or self-reflection is important for prevention. Awareness itself was sometimes described as empowering, helping participants step back from automatic social comparison. Lastly, a few participants noted that therapy could be helpful in general for preventing FoMO. This may reflect a broader recognition that FoMO is not only a behavioral issue (e.g., social media use) but also an emotional and cognitive one that benefits from deeper reflection and support.

*But when it comes to FoMO that appear even without this social media context, I think it’s just a realization that we’re going to miss out on something anyway, because there are so many things happening that choices are necessary. We can’t be everywhere, do everything, hang out with everyone. We just must make some decisions.* [Woman, 23, low FoMO]

#### Coping strategies.

Strategies for dealing with FoMO after it occurs are more common than those aimed at preventing it. These strategies are more frequently reported by individuals with high trait-FoMO. In contrast, people with low trait-FoMO often state that their FoMO is not intense enough to require any specific coping strategy. This suggests that for individuals with high trait-FoMO, FoMO may function as a recurring emotional challenge that requires active management, whereas for low trait-FoMO individuals, it may be more fleeting and easier to dismiss without intervention.

***Distraction.*** The most frequently mentioned strategy for managing FoMO is *distraction*. This is typically the first spontaneous response that comes to mind and is seen as an intuitive way to cope with the initial symptoms of FoMO. However, even participants who use distraction acknowledge that it provides only temporary relief and does not address the underlying issue in the long term. This positions distraction as an avoidant coping mechanism, which is effective for short-term relief but unlikely to reduce FoMO intensity or frequency over time. Participants have different methods of diverting their attention away from FoMO. For some, especially those with low trait-FoMO, distraction is the only strategy they have ever needed, as their experience of FoMO tends to be less intense. This may reflect that for individuals with lower FoMO sensitivity, behavioral diversion is sufficient to reduce emotional impact, without the need for deeper emotional processing. Distraction is a universal strategy mentioned for managing FoMO across various contexts. However, in the context of social media, a specific approach to distraction involved engaging in *more real-life activities* rather than virtual ones.

*I honestly see that distraction could help, although it’s a bit like ignoring these feelings, thoughts. I think that in the long run it wouldn’t be effective. Because it’s not like we’ll think through this anxiety, think about it, understand it. We’ll just do something else, something that will occupy our mind more, but this anxiety will come back sooner or later.* [Man, 24, low FoMO]

***Letting the feeling pass.*** Next intuitive strategy for dealing with FoMO, mentioned by some participants, is simply *letting the feeling pass*. They noted that, with time and understanding, the feeling of FoMO tends to fade. This approach is particularly common among individuals who experience FoMO with lower intensity.

***Self-reflection.*** Another strategy for dealing with FoMO is *self-reflection*. Participants described this technique as a way of understanding what FoMO is and how it might affect them, which requires a certain level of self-awareness. Self-reflection begins with recognizing that a particular feeling is, in fact, FoMO. Participants then try to rationalize and analyze their choices, realizing that their decisions align with their own wants and needs, and that the negative feelings stem from FoMO. This approach helps individuals distinguish between pressure caused by FoMO and a regular motivation or curiosity. Some noted that, over time, this technique can build self-confidence and provide reassurance. While self-reflection was mentioned more often in the context of offline FoMO, it could be applied universally, including in online settings. Its broader applicability suggests that it may be especially effective in contexts where FoMO is tied to life decisions, identity, or long-term goals.

***Focus on the present.*** A related approach tied to self-reflection is *focusing on the present*. This can be achieved through meditation, mindfulness techniques, or simply by concentrating on the benefits of the current moment, rather than dwelling on what one might be missing. These strategies reflect a shift toward emotion-focused regulation, helping individuals tolerate discomfort and re-anchor attention to the present rather than chasing imagined alternatives. Additionally, relaxation techniques similar to those used for managing stress were mentioned as potentially helpful in this context. This strategy was more commonly associated with offline FoMO.

*My strategy for dealing with FoMO is that if I can’t participate in something I really want to, and I end up doing something else, I tell myself, ‘Yes, I want to do what I’m doing now, not what I originally wanted to do; it doesn’t matter anymore.’ So it’s just tricking your brain into thinking you want to do what you’re doing now and focusing on that.* [Woman, 28, low FoMO]

***Online-related coping strategies.*** The next two strategies discussed are specific to dealing with FoMO in an online context. The first strategy is *limiting access to information*. Participants mentioned that after experiencing FoMO from something they saw online, they try to mitigate it by unfollowing certain accounts or uninstalling apps that cause discomfort. The goal is to reduce exposure to stimuli that might trigger negative emotions. However, some participants noted that this approach can sometimes have the opposite effect, as limiting access to information may intensify FoMO, especially for those already prone to it. A similar but more extreme strategy is a *social media detox*. In this case, participants mentioned using apps to limit their time on social media or stopping its use altogether for a period. Again, there is a risk that disconnecting from social media could backfire, leading people to think more about what they might be missing, thereby intensifying their FoMO. This illustrates the cyclic nature of FoMO regulation, where both engagement and avoidance can perpetuate the underlying anxiety, particularly in digital contexts. Both of these strategies, while helpful in the short term, only address the issue superficially.

In contrast, another strategy mentioned for managing online FoMO is *actively checking social media*. When participants felt FoMO about content online, they would check social media to ensure they were not missing out on anything. However, this approach can create a vicious cycle, where checking social media only heightens feelings of FoMO.

*I don’t know to what extent this detox solves the problem, because I would still have that feeling in the back of my mind that I want to know. So, on one hand, I’d be trying to limit the feeling of FoMO, but on the other, I’d still want to know* [Woman, 19, low FoMO]

***Support from others.*** Lastly, some participants mentioned that *talking to close friends or family* can be a helpful strategy for managing FoMO. This aligns with emotion-focused coping, particularly for offline FoMO experiences that involve relational disappointment or disconnection. In some cases, it may also serve as a form of distraction. However, participants noted that this strategy may be less effective if those close to them are also experiencing FoMO, as the conversation might not provide relief. In such cases, some participants suggested that undergoing *therapy* might be more beneficial, especially if FoMO symptoms are severe or accompanied by other issues. This suggests that for some individuals, FoMO may reflect broader emotional regulation challenges or self-worth issues that extend beyond specific situations.

In summary, most of the strategies discussed are applicable across various FoMO-related situations, with the exception of social media FoMO, which has two unique strategies specific to that context. These findings indicate that individuals use a range of coping strategies that differ in depth, duration, and function, ranging from quick behavioral fixes (e.g., distraction) to more reflective approaches (e.g., self-reflection, mindfulness). Notably, strategies linked to emotional awareness and self-reflection were more often used by individuals with higher psychological insight, suggesting that FoMO coping is shaped not only by intensity but also by emotional literacy and self-regulation capacity.

**Fig 2:**
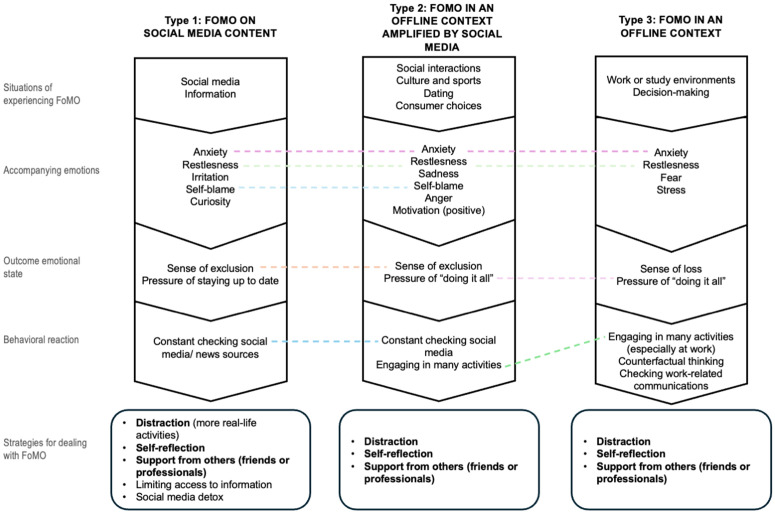
Overview of the results.

Fig 2 provides an overview of the results, combining different FoMO contexts with the emotions and strategies used to manage them. It is important to note that the themes presented in the figure are the most frequently mentioned; for example, irritation is not exclusive to FoMO on online content but is most characteristic of that context. Common themes across categories are connected by dashed lines, while shared strategies are highlighted in bold.

## Discussion

The aim of this study was to explore individuals’ experiences of FoMO across various contexts and the strategies they use to cope with it. The contexts in which FoMO is experienced were categorized into three groups: (1) FoMO related to social media content; (2) FoMO experienced in offline situations but amplified by social media; and (3) FoMO occurring solely in offline contexts. While each category reflected distinct situational triggers, the emotional landscape showed both shared and context-specific patterns. Notably, some emotional and behavioral reactions were common across contexts, such as anxiety and restlessness, while others were more prevalent in specific settings. This suggests that although FoMO may be a general psychological experience, its emotional intensity and expression are shaped by contextual cues and perceived stakes.

In the first category, FoMO related to social media content (Type 1), unique emotions included irritation and curiosity. This type of FoMO often resulted in feelings of exclusion and a pressure to stay up to date, with the primary behavioral response being constant social media checking. These findings align with previous research that links FoMO to increased social media use [1]. This may reflect a pattern of reactive, digitally reinforced behavior, where the fear of being uninformed leads to habitual checking.

For the second category – FoMO experienced offline but amplified by social media (Type 2) – the situations mentioned included social interactions, cultural and sports events, dating, and consumer choices. Unique emotional reactions in this context included sadness, anger, and even positive motivation, with self-blame being a common response, as it was in Type 1. While feelings of exclusion and frequent social media checking mirrored those in Type 1, the key difference was the pressure to “do it all” rather than just staying up to date. This often led to engaging in many activities as a direct result of FoMO. This form of FoMO seems to blur the boundary between aspiration and comparison, as individuals attempt to meet idealized social standards shaped by what others share online.

In the third category, where FoMO was experienced purely in offline contexts (Type 3), the situations typically involved work, study, or decision-making. Unique emotions in this context included fear and stress, with the outcome being a sense of loss. Like in Type 2, there was pressure to “do it all,” leading to increased activity. However, a unique response in this setting was counterfactual thinking. Checking work-related communications was also a behavioral response, similar to the social media checking seen in the first two categories, though here it involved more often professional or non-social media platforms. This category highlights a more introspective dimension of FoMO – one shaped less by visibility and more by internalized expectations and imagined alternatives.

The findings on the various situations where FoMO can be experienced are consistent with earlier research, particularly regarding situations commonly associated with FoMO [26,28–33]. Similarly, the most frequent emotional and behavioral reactions—such as anxiety, a sense of exclusion, and increased social media use—align with previous studies [1,3]. What is new, however, is the inclusion of multiple FoMO contexts and their emotional and behavioral reactions within a single in-depth study. This approach allows for more comprehensive comparisons, revealing both universal aspects of FoMO-related emotions and behaviors across contexts, as well as context-specific characteristics. This contextualized view of FoMO highlights how the phenomenon is not tied exclusively to digital life but reflects broader concerns related to social belonging, identity, and opportunity.

The exploration of strategies used to cope with FoMO was preceded by an examination of preventive techniques. The results suggest that respondents rarely use specific methods aimed at preventing FoMO. In the social media context, limiting social media use was the most commonly mentioned preventive measure. Outside of social media, the acceptance of FoMO as an inevitable part of life was noted, with this approach being more common among individuals with low trait-FoMO. These findings suggest that preventive efforts may be more intuitive and passive, particularly among those less affected, and that awareness alone may not be enough to trigger active prevention.

Regarding coping strategies, the findings indicate that most strategies are universal across different FoMO categories, with two exceptions that were specific to social media-related FoMO (type 1). In line with earlier studies, strategies such as limiting access to information or engaging in a total “social media detox” were commonly used for social media-related FoMO [11,12,35]. More general strategies that apply across all FoMO contexts include distraction (which varies depending on the context), self-reflection (such as mindfulness techniques that help individuals focus on the present and recognize their feelings of FoMO), and seeking support from others (either from close ones or professionals in more severe cases). While the strategies related to social media FoMO align with previous research, the broader, context-independent strategies represent a novel finding. Importantly, these strategies were not learned through formal interventions but emerged as natural responses, suggesting they may offer a practical foundation for designing relatable, low-barrier support tools. This focus on the strategies people intuitively use, rather than pre-planned interventions developed by professionals, is another unique contribution of the study.

Finally, when comparing individuals with high and low trait-FoMO, those with high trait-FoMO reported more intense negative emotions. In contrast, those with low trait-FoMO were more likely to acknowledge the positive aspects of FoMO, such as the motivation it can provide. Despite these emotional differences, both groups experienced FoMO in similar situations and employed similar coping strategies, although low-FoMO individuals more often mentioned that they felt no need for specific coping strategies at all. This suggests that trait-FoMO may not determine the kinds of strategies people use, but rather play a role in how necessary and emotionally urgent those strategies feel. It also underscores the importance of tailoring interventions to an individual’s level of FoMO sensitivity.

The current study has some limitations. While the in-depth qualitative approach provides valuable insights into individuals’ underlying thoughts and emotions, it does not quantify the prevalence of the observed patterns. Therefore, a quantitative follow-up study is recommended to measure these findings on a larger scale. Additionally, the study relied on self-reported data shared in one-on-one interviews, which may have been influenced by participants’ memory biases or hesitation to disclose certain experiences. Future research could address this by incorporating a mixed-methods approach to strengthen the reliability of the data. Another limitation concerns the demographic and cultural scope of the study. All participants were based in Poland, which may limit the generalizability of the findings to other cultural or social contexts. Norms around social interaction, digital behavior, and emotional expression can vary across cultures, potentially influencing how FoMO is experienced and managed. As such, future research should explore FoMO in diverse cultural settings to assess whether the emotional patterns and coping strategies identified here are consistent across populations.

Despite its limitations, this study offers several strengths. First, its in-depth qualitative approach allowed for a rich exploration of participants’ subjective experiences, capturing emotional nuance and behavioral complexity often missed by quantitative methods. Second, by examining FoMO across multiple contexts it contributes a more comprehensive understanding of how FoMO manifests in both online and offline settings. Third, the inclusion of individuals with varying levels of trait-FoMO enabled meaningful comparisons in emotional responses and coping styles. Finally, the study’s focus on intuitive, naturally occurring coping strategies provides practical insights that can inform the development of accessible and relatable interventions.

The study offers several theoretical contributions. First, the categorization of FoMO experiences aligns with Social Comparison Theory, as social comparisons appear to be a key factor underlying FoMO across all contexts. Regardless of the specific situation, these comparisons seem essential for FoMO to manifest. The findings also support the self-concept perspective proposed by Zhang et al. [22]. For instance, FoMO related to social media content (type 1) can be both social (e.g., staying up-to-date to avoid exclusion) and personal (e.g., staying informed to satisfy one’s curiosity). FoMO experienced offline but amplified by social media (type 2) tends to be more social, as it involves interactions and social events. In contrast, offline FoMO (type 3) is primarily personal, related to one’s individual choices and career experiences.

The current study has several practical implications. By examining FoMO in a broader context beyond social media, we uncover a wider range of experiences and emotions associated with this phenomenon. This broader perspective can inform various stakeholders, including mental health professionals, educators, and social media platforms, about the diverse manifestations of FoMO and its impact on individuals’ well-being. Moreover, understanding the intuitive coping strategies that people employ to manage FoMO can aid in promoting more effective interventions. Mental health practitioners can use this knowledge to develop tailored strategies that resonate with individuals’ existing coping mechanisms. For example, by incorporating distraction techniques, self-reflection practices such as mindfulness, and social support systems into therapeutic approaches, practitioners can enhance individuals’ resilience to FoMO. In clinical settings, recognizing and validating intuitive strategies can help clinicians build on clients’ natural coping styles, rather than attempting to replace them. In educational contexts, these insights could inform psychoeducation programs that teach students to identify FoMO, normalize its occurrence, and adopt adaptive responses, such as mindfulness, expectation management, or setting digital boundaries. Designing interventions around strategies people already use increases their relevance, accessibility, and long-term effectiveness. Future work could explore how intuitive coping strategies identified here could be adapted into formal interventions and tested for effectiveness. It may also be useful to examine whether different contexts of FoMO (e.g., academic, professional, or relational) benefit from targeted versus general coping approaches.

## Supporting information

S1 File Screening questionnaire. (PDF)

S2 File Discussion guide. (PDF)

S3 File Materials used in the study. (PDF)

S4 File Code tree. (PDF)
