# Supplementary material for: Many faces of FoMO: A qualitative in-depth investigation of context-specific experiences, emotions, and coping strategies
Source: PLoS One. 2025 Sep 2;20(9):e0330978. doi: 10.1371/journal.pone.0330978 (PMC12404441; doi:10.1371/journal.pone.0330978)
Supplement: S1 File — (PDF) [file pone.0330978.s001.pdf]

S1. Screening questionnaire

- **Demographics**

1. Age:
2. Gender:

- **FOMO Scale (Przybylski et al., 2013)**

Below is a collection of statements about your everyday experience. Using the scale provided please indicate how true each statement is of your general experiences. Please answer according to what really reflects your experiences rather than what you think your experiences should be. Please treat each item separately from every other item.

Not at all true of me | 1
Slightly true of me | 2
Moderately true of me | 3
Very true of me | 4
Extremely true of me | 5

1. I fear others have more rewarding experiences than me.
2. I fear my friends have more rewarding experiences than me.
3. I get worried when I find out my friends are having fun without me.
4. I get anxious when I don't know what my friends are up to.
5. It is important that I understand my friends "in jokes."
6. Sometimes, I wonder if I spend too much time keeping up with what is going on.
7. It bothers me when I miss an opportunity to meet up with friends.
8. When I have a good time it is important for me to share the details online (e.g. updating status).
9. When I miss out on a planned get-together it bothers me.
10. When I go on vacation, I continue to keep tabs on what my friends are doing
