# Supplementary material for: Many faces of FoMO: A qualitative in-depth investigation of context-specific experiences, emotions, and coping strategies
Source: PLoS One. 2025 Sep 2;20(9):e0330978. doi: 10.1371/journal.pone.0330978 (PMC12404441; doi:10.1371/journal.pone.0330978)
Supplement: S2 File — (PDF) [file pone.0330978.s002.pdf]

S2. Discussion guide

1. **Welcome, presentation of rules**
2. **Warm-up (social media use)**
   - What social media platforms do you use?
   - How often do you use social media? How much time do you spend there?
   - Is social media important to you? Why yes/no?
   - Look at the emotion diagram below [Plutchik's wheel of emotions]. Think about what emotions you experience when using social media.
3. **FoMO – definition and introduction**
   - Have you heard of the phenomenon of FOMO ('fear of missing out')? If so, try to describe in your own words what FOMO is.
   - When you hear 'FOMO', what does it suggest?
   - Definition of FoMO read by the moderator.
   - Projection technique exercise: persona with FoMO and without FoMO.
4. **Experiencing FoMO**
   - Do you experience FOMO? [Only if the answer is yes] How often do you experience FOMO?
   - Think back to the last time you felt FOMO. What was it? What did you feel then?
   - How do you know if the feeling you're experiencing is FOMO?
   - In what types of situations do you experience FoMO? What emotions are associated with it then?
   - First, respondents freely generate examples, and then the moderator ask about the following areas in general:
     a. Shopping and decisions
     b. Work/career/studies
     c. Social relationships/social life
5. **Coping with FoMO**
   - Do you take any actions to counteract the feeling of FOMO? If so, what are these actions?
   - If you do feel FOMO, how do you deal with it? What actions do you take to combat it/ to feel better?
   - First, respondents freely generate examples, and then the moderator ask about the following areas in general:
     a. Limiting social media use ('detox')
     b. Meditation/ mindfulness
     c. Distraction (doing something else)
     d. Talking to loved ones
     e. Limiting the flow of information
6. **General reflections, thanks and ending**
