# Supplementary material for: Many faces of FoMO: A qualitative in-depth investigation of context-specific experiences, emotions, and coping strategies
Source: PLoS One. 2025 Sep 2;20(9):e0330978. doi: 10.1371/journal.pone.0330978 (PMC12404441; doi:10.1371/journal.pone.0330978)
Supplement: S3 File — (PDF) [file pone.0330978.s003.pdf]

S3. Materials used in the study.

a) Plutchik's wheel of emotions (the Polish version was used in the study).

Due to copyright restrictions, the original image used in this study cannot be published or distributed publicly. However, the exact diagram shown to participants is available upon request to the author.

b) Projection technique exercise.

Instruction: *The following exercise requires you to use your imagination. In a moment, you'll see photos of different people. From these photos, choose 1 portrait of a person who you think has strong FoMO and 1 portrait of someone who doesn't usually have FoMO. Think about who these people are. What are their names? What do they do? What are their personality traits? How do they spend their free time? What do you think about them? Would you like these people?*

Due to copyright restrictions, the original images used in this study cannot be published or distributed publicly. However, the exact photos shown to participants (both men and women) are available upon request to the author.
