# Supplementary material for: Many faces of FoMO: A qualitative in-depth investigation of context-specific experiences, emotions, and coping strategies
Source: PLoS One. 2025 Sep 2;20(9):e0330978. doi: 10.1371/journal.pone.0330978 (PMC12404441; doi:10.1371/journal.pone.0330978)
Supplement: S4 File — (PDF) [file pone.0330978.s004.pdf]

S4. Code tree.

CODE	FREQUENCY
**Code tree**	**653**
**Perception of FoMO**	0
**Spontaneous mentions**	11
**Initial associations**	14
Fear of exclusion	8
Social media	5
Social comparisons	2
Addictions	2
Social gatherings	2
Information noise	1
**Initial associations - Emotions**	0
Negative	0
Sadness	9
Anxiety	9
Irritation	8
Vigilance/ awaiting	4
Other	6
Positive	0
Curiosity	1
**Associations with FoMO (projection technique exercise)**	0
Persona without FoMO	35
Individual traits	8
Optimism	9
Openness to others	7
Independence from others' opinions	7
Self-confidence	1
Work and interests	21
Persona with FoMO	37
Work and interests	20
Individual traits	9
Need to impress others	10
Many activities	7
Workaholism	6
Social comparisons	4
Sadness	3
Low self-esteem	3
Unfulfilled	2
Sense of loss	1
Evaluation of the personas	23
**Experiencing FoMO**	0
**Frequency**	15
Lowering the frequency	3
**Situations of experiencing FoMO**	0
Work/ study	23
Social interactions	19
Social media	17
Consumer choices	9

Decisions	9
Information	9
Culture/ sport	7
Dating	6
No FOMO experiences	3
Other	6
**Observing the experiences of other people**	10
**Emotions**	0
**Negative**	3
Anxiety	7
Restlessness	6
Self-blame	6
Sadness	6
Sense of exclusion	5
Motivation (pressure)	5
Sense of loss	5
Irritation	4
Stress	4
Anger	3
Other	3
**Positive**	0
Motivation (positive)	5
Curiosity	3
Relief	2
Other	3
**Strategies of dealing**	0
Distraction	23
More real-life activities	4
Self-reflection	18
Limiting access to information	14
Talking to close friends and family	10
Social media detox	10
Focusing on the present	9
Therapy	4
Checking social media	4
Letting the feeling pass	4
No need to deal with FoMO	2
Other	2
**Prevention methods**	0
Selectively choosing social media content	3
Acceptance	3
Knowledge	2
Lack of expectations	2
Therapy	2
Social media limits	2
Other	2
**Reflections about FoMO**	11
